# An *in vitro* co-culture model with CAR-T cells, antigen-presenting cells, and tumor cells to evaluate CAR-T cell-induced cytokine release syndrome

**DOI:** 10.3389/fcimb.2026.1721114

**Published:** 2026-01-26

**Authors:** Yuke Ren, Zhi Lin, Shuangxing Li, Ruiqiu Zhang, Zixuan Lai, Hua Jiang, Zhe Qu, Guitao Huo, Di Zhang, Yanwei Yang, Bo Li, Xingchao Geng

**Affiliations:** 1 National Institutes for Food and Drug Control, Chinese Academy of Medical Sciences and Peking Union Medical College, Beijing, China; 2 National Center for Safety Evaluation of Drugs, Key Laboratory of Beijing for Quality control and Non-clinical Research and Evaluation for Cellular and Gene Therapy Medicinal Products, National Institutes for Food and Drug Control, Beijing, China; 3 State Key Laboratory of Natural Medicines, China Pharmaceutical University, Nanjing, China; 4 Institute for Biological Product Control, National Institutes for Food and Drug Control, Beijing, China

**Keywords:** CAR-T cells, CRS, cytokine release syndrome, *in vitro* assay, non-clinical safety evaluation

## Abstract

**Objective:**

Chimeric antigen receptor (CAR) T-cell therapy has demonstrated remarkable efficacy in hematological malignancies. However, it can also cause severe systemic toxicity, known as cytokine release syndrome (CRS). Therefore, the potential of CAR-T cells to cause toxicity *in vivo* should be evaluated in preclinical models prior to first-in-human trials. Although murine models exist for this purpose, they are typically complex xenograft systems available only to a limited number of laboratories. Therefore, development of an *in vitro* assay to assess CRS elicited by CAR-T cells is warranted.

**Methods:**

CAR-T cells, macrophages, or immature dendritic cells (iDCs), along with tumor target cells, were co-cultured under different conditions. The release of CRS-related cytokines, IFN-γ and IL-6, was measured to simulate cytokine release during CAR-T-induced CRS. Additionally, the cellular source of the key CRS cytokine IL-6 was investigated.

**Results:**

A co-culture system containing only CAR-T cells and tumor cells failed to recapitulate the key feature of CRS, specifically a significant elevation of IL-6. However, when CAR-T cells were co-cultured with antigen-presenting cells (macrophages or iDCs) and tumor cells, the core CRS cytokine IL-6 was significantly elevated in an *in vitro* cell culture model, indicating that this system effectively mimics cytokine release during CAR-T-induced CRS. Furthermore, macrophages and iDCs are the primary cellular sources of IL-6 during CRS, with macrophages playing a central role in the development of CRS. Additionally, a co-culture system involving CAR-T cells, tumor cells, and macrophages under these conditions can indicate the occurrence of clinically severe-grade CRS.

**Conclusion:**

Macrophages and iDCs play a critical role in the development of CAR-T therapy-induced CRS. The triple-cell co-culture system, comprising CAR-T cells, macrophages or iDCs, and tumor cells, provides a viable *in vitro* model for assessing CAR-T cell-induced CRS.

## 1 Introduction

Chimeric antigen receptor (CAR) T-cell therapy has demonstrated significant clinical efficacy in the treatment of certain hematological malignancies. To date, seven CAR-T products have been approved by the U.S. FDA and six by the EMA for indications including relapsed or refractory diffuse large B-cell lymphoma (Halford et al., 2021; Neelapu et al., 2017), acute B-cell lymphoblastic leukemia (Maude et al., 2018; Roddie et al., 2024), mantle cell lymphoma (Wang et al., 2020), follicular lymphoma (Fowler et al., 2022), multiple myeloma (San-Miguel et al., 2023), and chronic lymphocytic leukemia (Siddiqi et al., 2023). Thus, CAR-T therapy represents a major advancement in the treatment of hematologic cancers. CAR-T therapy has thus brought new hope to patients with hematologic cancers. However, associated adverse effects, particularly cytokine release syndrome (CRS), can in severe cases be life-threatening.

**Figure 1:**
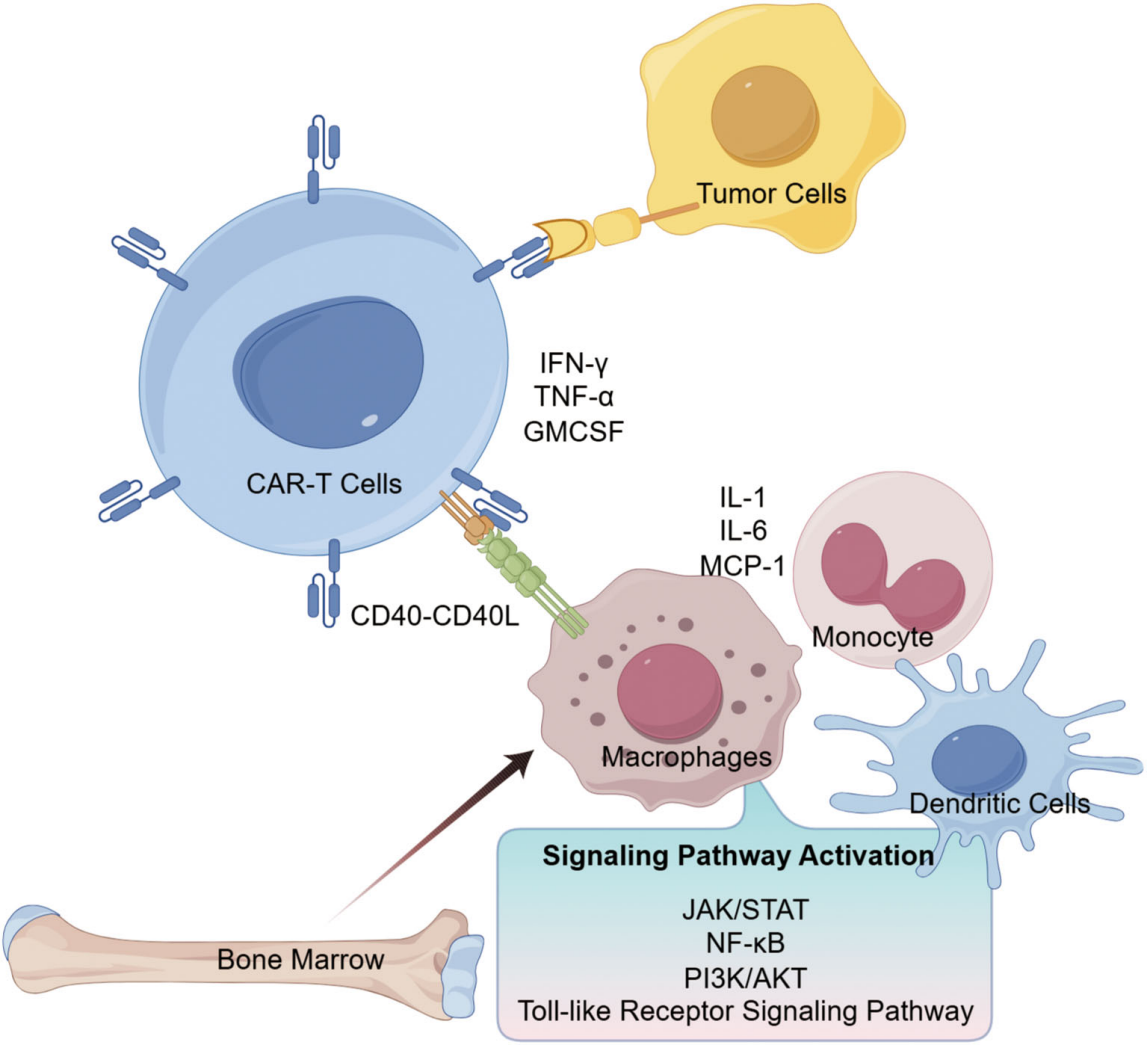
Key cytokines and signaling pathways in CRS induced by CAR-T cell therapy (By Figdraw).

The mechanism underlying CAR-T cell-induced CRS is illustrated in **Figure 1**. Upon engagement with tumor-associated antigens, CAR-T cells release inflammatory factors such as IFN-γ and GM-CSF. These cytokines stimulate immune and non-immune cells to further secrete IL-1, IL-6, IL-10, IFN-γ, TNF-α, and MCP-1, which in turn amplify T-cell and immune activation, establishing a positive feedback loop. This cascade leads to excessive systemic cytokine levels and the onset of CRS (Schroeder et al., 2024; Majumder, 2023). Additionally, receptor–ligand interactions (e.g., CD40–CD40L) between CAR-T cells and monocytes/macrophages contribute to monocyte lineage activation (Giavridis et al., 2018). The pathophysiology of CRS also involves multiple signaling pathways, including JAK-STAT and NF-κB, which when activated promote inflammatory responses (Wei et al., 2022). As CRS represents a major toxicity of CAR-T treatment, preclinical assessment of CRS risk is essential before initiating clinical trials.

In preclinical evaluation of CAR-T products, immunodeficient mice bearing human tumor xenografts are commonly used. However, due to interspecies differences—such as the inability of IFN-γ and IL-6 to be recognized across species (Giavridis et al., 2018) —and the lack of a complete immune microenvironment in immunodeficient mice (Zhao et al., 2019), it is difficult to accurately simulate the cytokine release syndrome (CRS) that occurs in humans. There is therefore a need to establish *in vitro* models for assessing the potential CRS risk of CAR-T cells. Growing evidence indicates that CRS is driven by interactions between CAR-T cells and endogenous myeloid cells, such as macrophages or dendritic cells. In this study, we developed an *in vitro* co-culture system of three types of cell, which includes CAR-T cells, macrophages or iDCs, and tumor cells, aiming to directly replicate the key interactions between CAR-T cells and human myeloid cells in patients, providing a more relevant platform for predicting CRS.

## 2 Materials and methods

### 2.1 Cells

CD19 CAR-T cells were provided by the Cell Bank of the National Institutes for Food and Drug Control (NIFDC). The cells were activated and expanded using ImmunoCult™-XF T Cell Expansion Medium (STEMCELL Technologies) containing ImmunoCult™ Human CD3/CD28 T Cell Activator (STEMCELL Technologies) and recombinant human IL-2 (Beijing Kexin Protein Biotechnology Co., Ltd.). Nalm6-CBG cells were provided by Toxicology Division of National Center for Safety Evaluation of Drugs of NIFDC. The cells were cultured in RPMI 1640 medium (Solarbio) supplemented with 10% fetal bovine serum (FBS, Gibco) and 1% penicillin-streptomycin (Solarbio). Human immature dendritic cells (iDCs) (Lot. 2311140160) and macrophages (M1) (Lot. 2310250152) were obtained from SCHBIO BIOTECH Co., Ltd. and maintained in RPMI 1640 medium (Solarbio) containing 10% heat-inactivated FBS (Gibco) and 1% penicillin-streptomycin (Solarbio).

### 2.2 Antibodies and reagents

Recombinant human IL-4, GM-CSF, and TNF-α for dendritic cell induction were purchased from Beijing Kexin Protein Biotechnology Co., Ltd. Antibodies against CD80 and CD86 for phenotyping dendritic cells were obtained from BD Biosciences. D-Luciferin for *in vivo* imaging was sourced from Absin Biotechnology Co., Ltd. Human IFN-γ and IL-6 ELISA kits were procured from Absin Biotechnology Co., Ltd. Immunofluorescence antibodies for CD3, HLA-DR, and IL-6 were acquired from Abcam. Anti-fade mounting medium with DAPI was purchased from Solarbio.

### 2.3 *In vitro* cytotoxicity assay

For the cytotoxicity assay of CAR-T cells, CAR-T cells (calculated based on the CAR^+^ population) and Nalm6-CBG tumor cells were used at effector-to-target (E:T) ratios of 5:1, 10:1, and 20:1. The cells were incubated for 72 hours in a total volume of 200 μL of RPMI 1640 medium supplemented with 10% heat-inactivated FBS. After incubation, supernatants were collected for further analysis, and the luciferase activity of remaining tumor cells was measured by the IVIS Spectrum CT imaging system.

### 2.4 *In vitro* cytotoxicity assay of tumor cells by CAR-T cells co-cultured with tumor antigen-loaded dendritic cells

**Figure 2:**
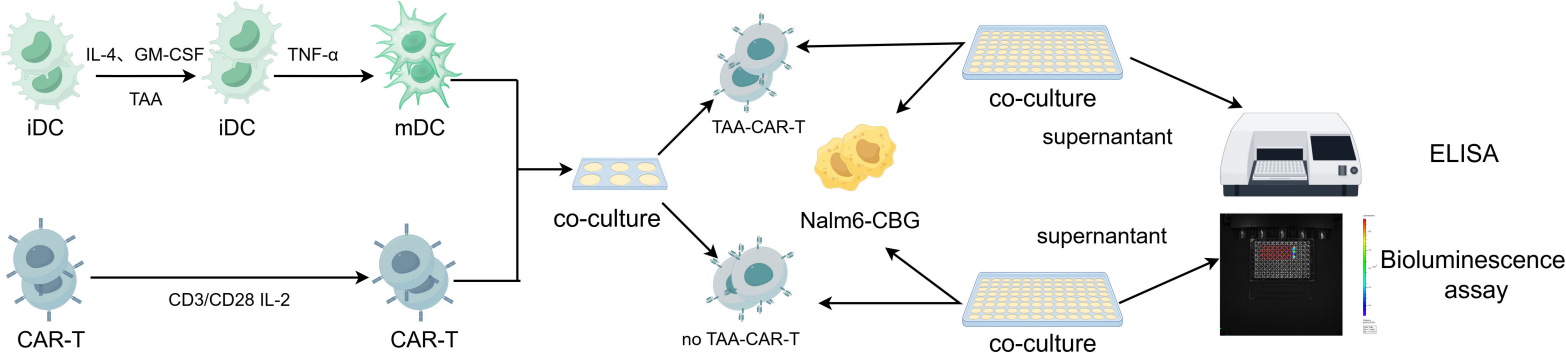
Experimental flowchart of *in vitro* cytotoxicity assay of tumor cells by CAR-T cells co-cultured with tumor antigen-loaded dendritic cells (By Figdraw). iDCs were matured with 500 U/mL IL-4, 1000 U/mL GM-CSF, with or not 10 μg/mL TAA, and 500 U/mL TNF-α, then co-cultured with CAR-T cells at a 1:10 ratio for 72 h. Subsequently, cytotoxicity assay was performed at E:T ratios of 5:1, 10:1, and 20:1; supernatants were collected for cytokine analysis, and residual tumor cells were subjected to *in vitro* bioluminescence assay. TAA: tumor-associated antigen.

Routinely cultured Nalm6-CBG cells were collected and adjusted to a density of 5 × 10^6^ cells/mL in sterile cryovials. The cell suspension was subjected to four complete cycles of freeze-thaw treatment (between −196°C and 37°C). The lysate was centrifuged at 12,000 rpm for 5 min, and the supernatant was collected. The protein concentration of the tumor antigen was determined using a BCA protein assay kit (Thermo Fisher Scientific). iDCs were cultured for 24 h in RPMI 1640 medium with 10% heat-inactivated FBS supplemented with 500 U/mL IL-4, 1000 U/mL GM-CSF, 1 μg/mL tumor-associated antigen (TAA). To induce DC maturation, 500 U/mL TNF-α was then added and the cells were cultured for an additional 48 h. CAR-T cells were co-cultured with the matured DCs at a ratio of 10:1 for 72 h. The CAR-T cells were then harvested and co-cultured with tumor cells at E:T ratios of 5:1, 10:1, and 20:1 for 72 h. Following this incubation, supernatants were collected for analysis, and the cells were subjected to *in vitro* bioluminescence assay. The experimental workflow is illustrated in **Figure 2**.

### 2.5 Co-culture of CAR-T cells, macrophages/iDCs, and tumor cells

CD19 CAR-T cells (calculated based on CAR^+^ population), iDCs or M1 macrophages, and tumor cells were adjusted to a density of 2 × 10^6^ cells/mL. These three types of cells were co-cultured at a ratio of 2:1:1 in a total volume of 200 μL of RPMI 1640 medium supplemented with 10% heat-inactivated FBS for 48 hours. A control group consisting of only CAR-T cells and tumor cells (two-cell co-culture) was set up in parallel. After incubation, supernatants were collected for analysis, and the cells were subjected to *in vitro* bioluminescence assay.

### 2.6 *In vitro* bioluminescence assay

Tumor cells lysed with lysis buffer were used as the positive control. The plate was centrifuged, and the supernatant was removed. A working solution of D-luciferin was prepared at a concentration of 300 μg/mL in RPMI 1640 medium, and 100 μL was added to each well. After incubation at 37°C for 30 minutes, *in vitro* bioluminescence assay was performed using the IVIS Spectrum CT imaging system.

### 2.7 Cytokines detection by ELISA

Supernatants from the cell co-cultures were collected and diluted, and the concentrations of human IFN-γ and IL-6 were measured using enzyme-linked immunosorbent assay (ELISA) according to the manufacturer’s instructions.

### 2.8 Immunofluorescence

For immunofluorescence analysis, co-cultured cells of three types cells were fixed with 4% tissue fixative, washed twice with PBS, and blocked with goat serum for 1 hour. The cells were then incubated overnight at 4°C with the following primary antibodies: rabbit anti-IL-6 (1:50 dilution) and mouse anti-CD3 (1:500 dilution), along with a directly conjugated antibody Alexa Fluor 555 anti-HLA-DR (1:250 dilution). Subsequently, the cells were stained with secondary antibodies Alexa Fluor 488 anti-rabbit (1:500) and Alexa Fluor 647 anti-mouse (1:500). Finally, the samples were mounted with anti-fade mounting medium containing DAPI and sealed. Imaging was performed using a high-content cell imaging system.

### 2.9 Data analysis

All data were analyzed using GraphPad Prism 10. Where appropriate, either one-way ANOVA or t-test was employed. Data are presented as mean ± standard deviation. For ANOVA, P values were determined using Dunnett’s multiple comparison test.

## 3 Results

### 3.1 No IL-6 was detected during CAR-T cell-mediated killing of target cells

**Figure 3:**
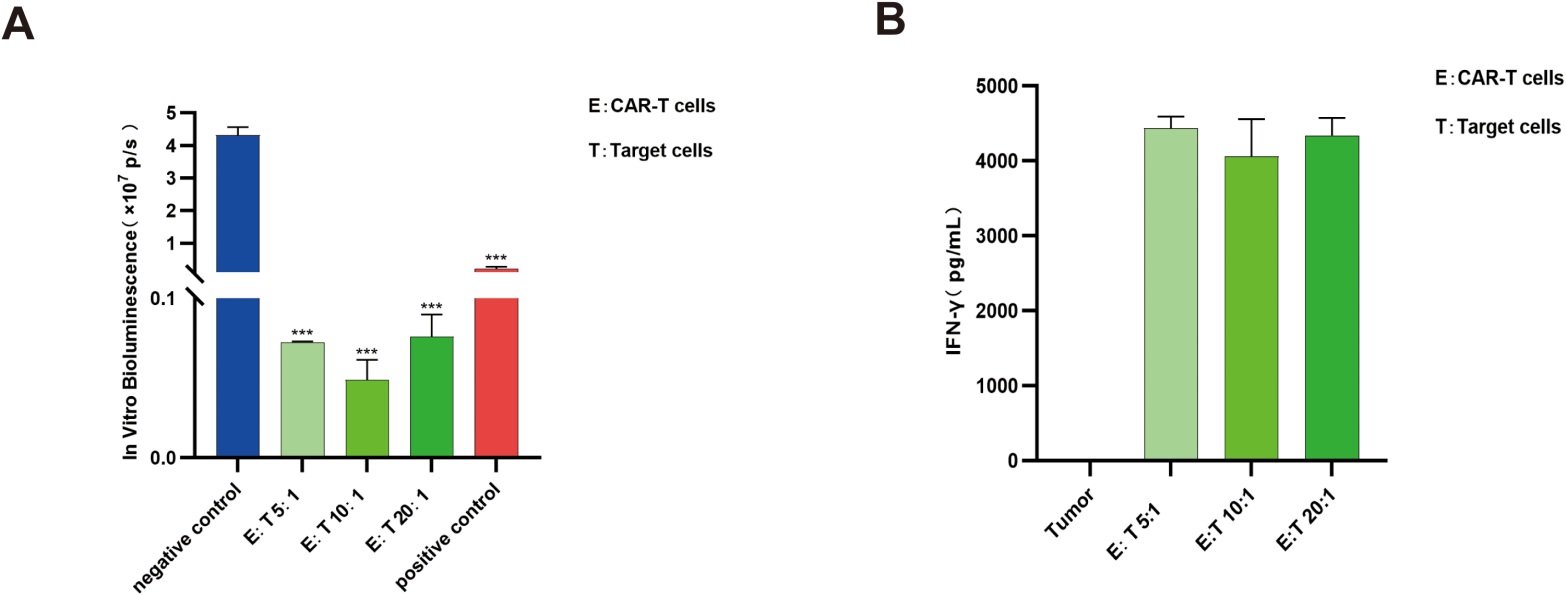
CAR-T cells demonstrated significant cytotoxicity, accompanied by elevated IFN-γ levels during tumor cell elimination. **(A)** Compared with the negative control group, CAR-T cells exhibited substantial cytotoxicity at E:T ratios of 5:1, 10:1, and 20:1 (***P < 0.001); n=3. **(B)** IFN-γ levels were significantly increased during CAR-T cell-mediated tumor cell killing; n=3.

The *in vitro* pharmacodynamic evaluation of CAR-T cells typically involves co-culturing CAR-T cells with target cells to assess cytotoxic activity. During the targeted engagement with tumor cells, CAR-T cells release cytokines such as IFN-γ, which can further enhance their antitumor efficacy. To investigate whether IL-6 is secreted during CAR-T cell-mediated killing, we co-cultured CAR-T cells with target cells and evaluated cytotoxicity using *in vitro* bioluminescence assay of tumor cells, while measuring IFN-γ and IL-6 levels in the supernatant by ELISA. The results demonstrated significant tumor cell killing by CAR-T cells at E:T ratios of 5:1, 10:1, and 20:1 (p>0.05) (**Figure 3A**), accompanied by markedly elevated IFN-γ levels under all three conditions (**Figure 3B**). However, no IL-6 was detected in the co-culture of two types of cells system at any of the E:T ratios tested.

### 3.2 IL-6 secretion detected during CAR-T cell-mediated killing following antigen presentation by dendritic cells

#### 3.2.1 Culture and characterization of dendritic cells

**Figure 4:**
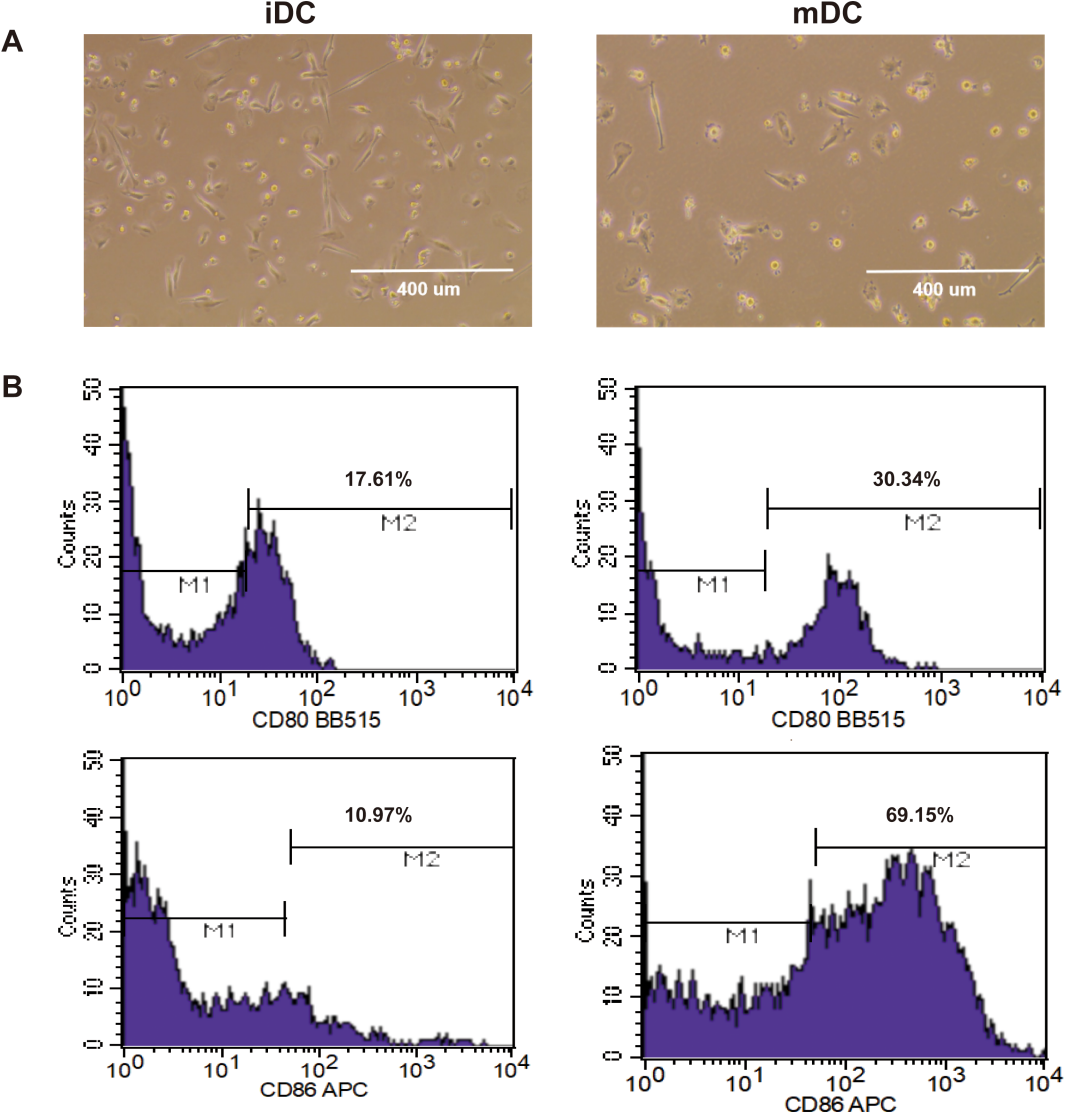
iDCs differentiated into mDCs following induction with IL-4, GM-CSF, and TNF-α. **(A)** iDCs exhibited irregular morphologies such as spherical and spindle shapes (left), while extensive dendritic projections extended from their surfaces after induction (right). **(B)** Following induction, iDCs (left) matured into mDCs (right), showing elevated surface expression of CD80/CD86. iDC: immature dendritic cells; mDC: mature dendritic cells.

Dendritic cells (DCs) are the most potent professional antigen-presenting cells (APCs) in the human body, capable of capturing and processing tumor antigens, then presenting them to T cells to initiate T-cell activation. iDCs were cultured with IL-4, GM-CSF, and Nalm6-CBG tumor antigen (TAA-DC group) or with IL-4 and GM-CSF only (no TAA-DC group) for 48 hours. At this stage, the iDCs exhibited irregular morphologies, including round and spindle shapes. After the addition of TNF-α and further culture for 24 hours, the DCs matured and displayed extensive dendritic protrusions (**Figure 4A**). Concurrently, mature DCs showed upregulated expression of the co-stimulatory molecules CD80 and CD86 (**Figure 4B**).

#### 3.2.2 Elevated IL-6 levels during target cell killing by CAR-T cells following co-culture with dendritic cells

**Figure 5:**
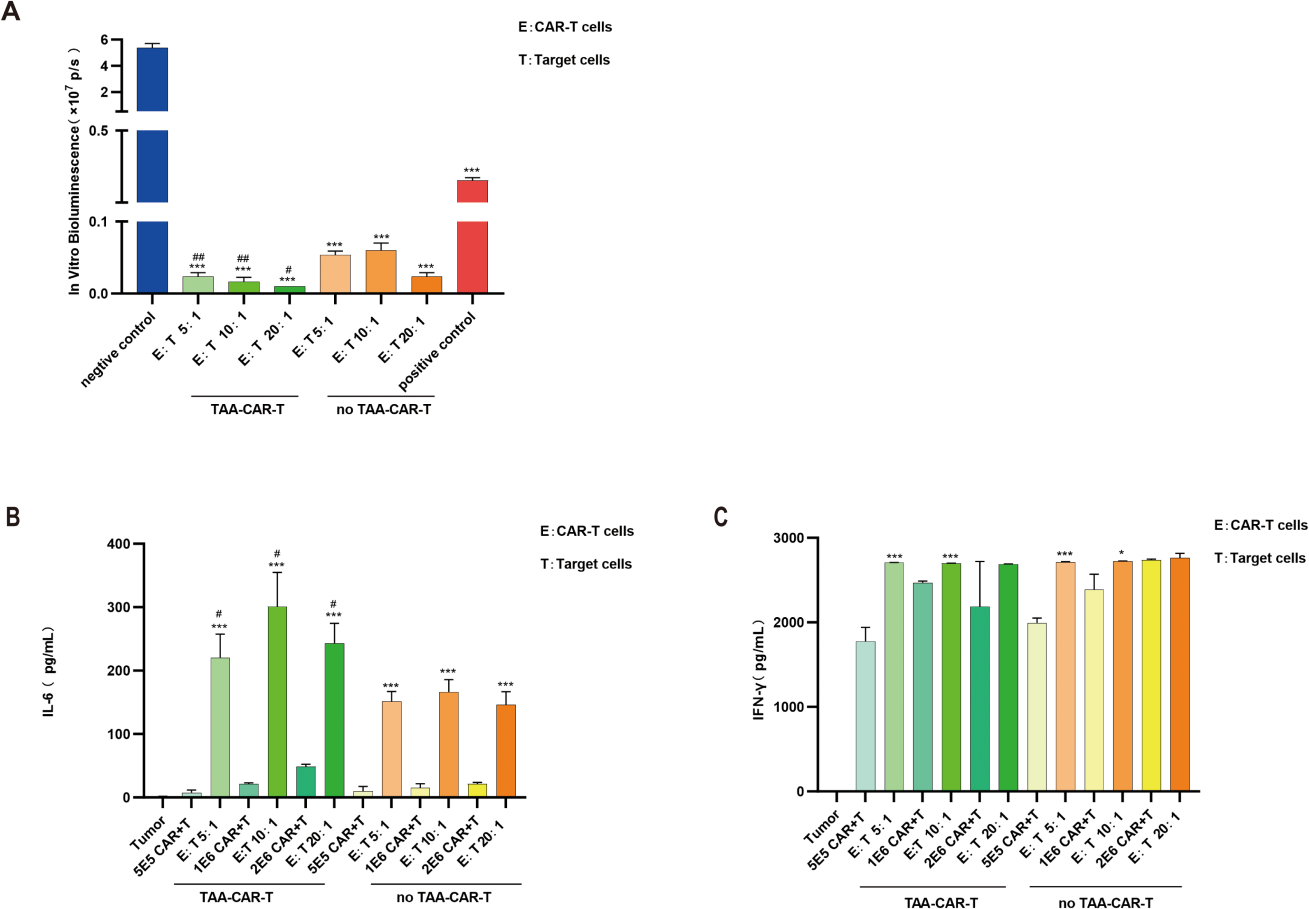
Tumor cell killing by CAR-T cells co-cultured with DCs loaded with or without TAA. **(A)***In vitro* bioluminescence of tumor cells. Compared with the negative control group, both TAA-CAR-T cells and no TAA-CAR-T cells demonstrated significant cytotoxicity at E:T ratios of 5:1, 10:1, and 20:1 (***, *P* < 0.001). The cytotoxicity of TAA-CAR-T cells was stronger than that of no TAA-CAR-T cells (#*P* < 0.05; ##*P* < 0.01); n=3. **(B)** Levels of IL-6. Both TAA-CAR-T cells and no TAA-CAR-T cells showed significantly elevated IL-6 levels during tumor cell killing (*P < 0.001), and the IL-6 level in the TAA-CAR-T group was significantly higher than that in the no TAA-CAR-T group (#, *P* < 0.05); n=3. **(C)** Levels of IFN-γ. Both TAA-CAR-T cells and no TAA-CAR-T cells exhibited increased IFN-γ levels during tumor cell killing (*P < 0.05; ***P < 0.001). No significant difference was observed between the TAA-CAR-T and no TAA-CAR-T groups; n=3. TAA, tumor-associated antigen; TAA-CAR-T, CAR-T cells pre-cultured with TAA-loaded dendritic cells; no TAA-CAR-T, CAR-T cells pre-cultured with dendritic cells not loaded with TAA.

To investigate whether tumor antigen-loaded DCs influence cytokine secretion during CAR-T-mediated tumor cell killing, DCs were first pulsed with tumor antigen and matured *in vitro*, then co-cultured with CAR-T cells for 72 hours (generating TAA-CAR-T and no TAA-CAR-T groups). These primed CAR-T cells were subsequently incubated with tumor cells at E:T ratios of 5:1, 10:1, and 20:1 for 72 hours. *In vitro* bioluminescence measurements revealed that both TAA-CAR-T and no TAA-CAR-T cells exhibited potent cytotoxic activity against tumor cells. However, TAA-CAR-T cells demonstrated significantly enhanced killing capacity compared to no TAA-CAR-T cells following antigen presentation. Previous studies have suggested that infusion of antigen-expressing cells can activate and promote CAR-T cell expansion (Davila et al., 2014). Our findings indicate that CAR-T cells co-cultured with antigen-pulsed DCs (TAA-CAR-T) exhibited superior cytotoxicity across all E:T ratios compared to no TAA-CAR-T cells (p>0.05) (**Figure 5A**).

To examine cytokine secretion during tumor cell killing by CAR-T cells following co-culture with APCs, supernatants were collected after 72 hours of co-culture with tumor cells. Results demonstrated that both TAA-CAR-T and no TAA-CAR-T cells secreted high levels of IFN-γ alone, along with minimal IL-6 secretion. However, during tumor cell killing, both IL-6 and IFN-γ levels were significantly elevated (p>0.05) (**Figures 5B, C**). These findings indicate that CAR-T cells can secrete IFN-γ, and its production increases during tumor cell engagement. IFN-γ can serve as a marker for evaluating CAR-T effector function, and in our experiments, substantial IFN-γ secretion was observed even at the 5:1 E:T ratio.

IL-6 is a cytokine secreted by various immune cells, including DCs that plays crucial roles in immunity and inflammation. In this study, minimal IL-6 was detected in supernatants from CAR-T cells co-cultured with either TAA-loaded or unloaded DCs (TAA-CAR-T or no TAA-CAR-T alone). However, during tumor cell killing, IL-6 levels increased substantially, with TAA-CAR-T cells producing significantly more IL-6 than no TAA-CAR-T cells (p>0.05)(**Figure 5B**). These results demonstrate that APCs play a critical regulatory role in CRS pathogenesis, particularly in modulating IL-6 secretion.

### 3.3 The core cytokine IL-6 of CRS is significantly elevated in CAR-T cell co-culture with APCs and target cells

IFN-γ is a key cytokine mediating the cytotoxic function of CAR-T cells (Huang et al., 2023); however, excessively high IFN-γ levels can contribute to the development of CRS. IL-6 serves as a core cytokine in CRS pathogenesis (Yang et al., 2023) and is secreted by macrophages and dendritic cells, among other cell types. To establish an *in vitro* model for evaluating CAR-T-induced CRS, this study employed a triple-cell co-culture system containing CAR-T cells, APCs (macrophages or iDCs), and tumor cells at a 2:1:1 ratio. Control groups included CAR-T cells co-cultured with tumor cells at a 2:1 ratio and tumor cells cultured alone. After 48 hours of incubation, supernatant levels of IFN-γ and IL-6 were measured.

**Figure 6:**
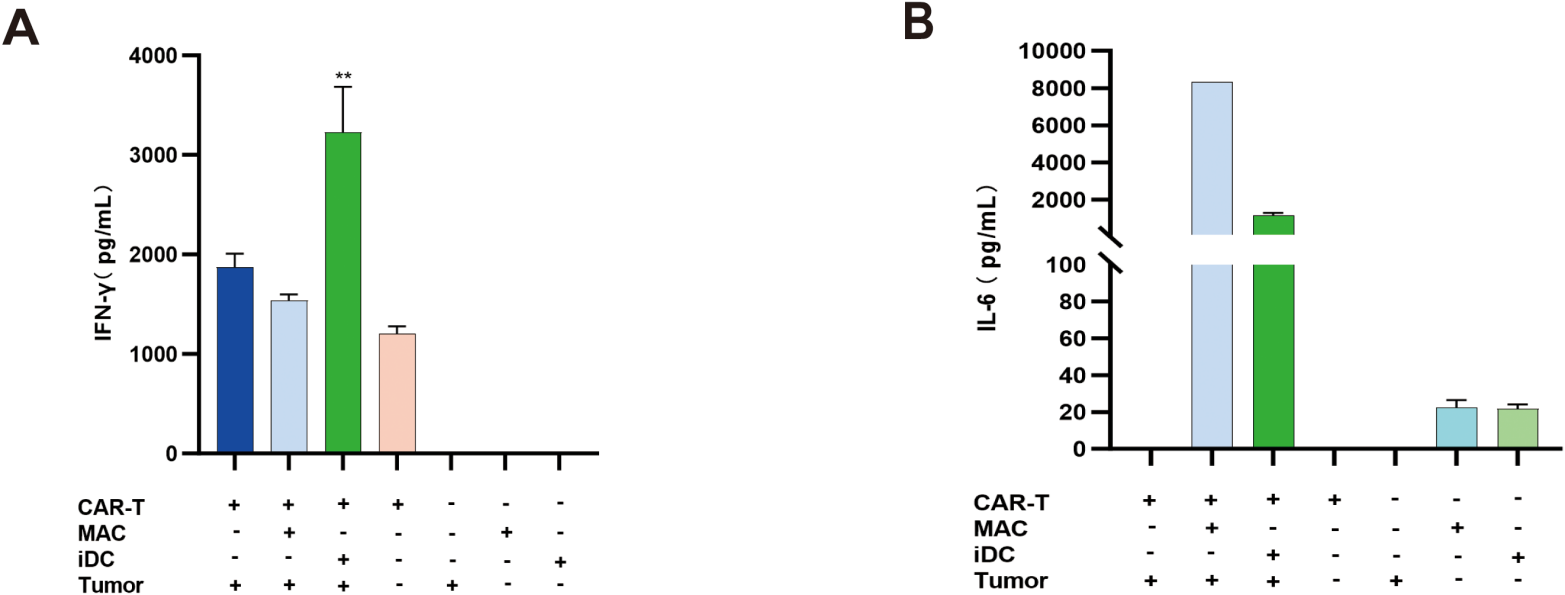
Cytokine expression following co-culture of CAR-T cells, macrophages/iDCs, and tumor cells. **(A)** IFN-γ levels under different culture conditions. The triple-cell co-culture of CAR-T cells, iDCs, and tumor cells resulted in a significant increase in IFN-γ levels (**, *P* < 0.01); n=3. **(B)** IL-6 levels under different culture conditions. Both macrophages and iDCs significantly elevated IL-6 levels in the co-culture system, with macrophages playing a more substantial role in the increase of IL-6; n=3.

Results demonstrated that while macrophages did not significantly influence IFN-γ levels in the co-culture system, the inclusion of iDCs markedly enhanced IFN-γ secretion (**Figure 6A**). In contrast, both macrophages and iDCs substantially elevated IL-6 levels, with macrophages exerting the most pronounced effect on IL-6 production (**Figure 6B**). These findings indicate that in CAR-T-induced CRS, iDCs may play a critical regulatory role in IFN-γ secretion, whereas macrophages serve as the primary cellular source of IL-6. Furthermore, different APCs appear to distinctly modulate the secretion of specific cytokines.

### 3.4 IL-6 primarily originates from APCs during CRS

**Figure 7:**
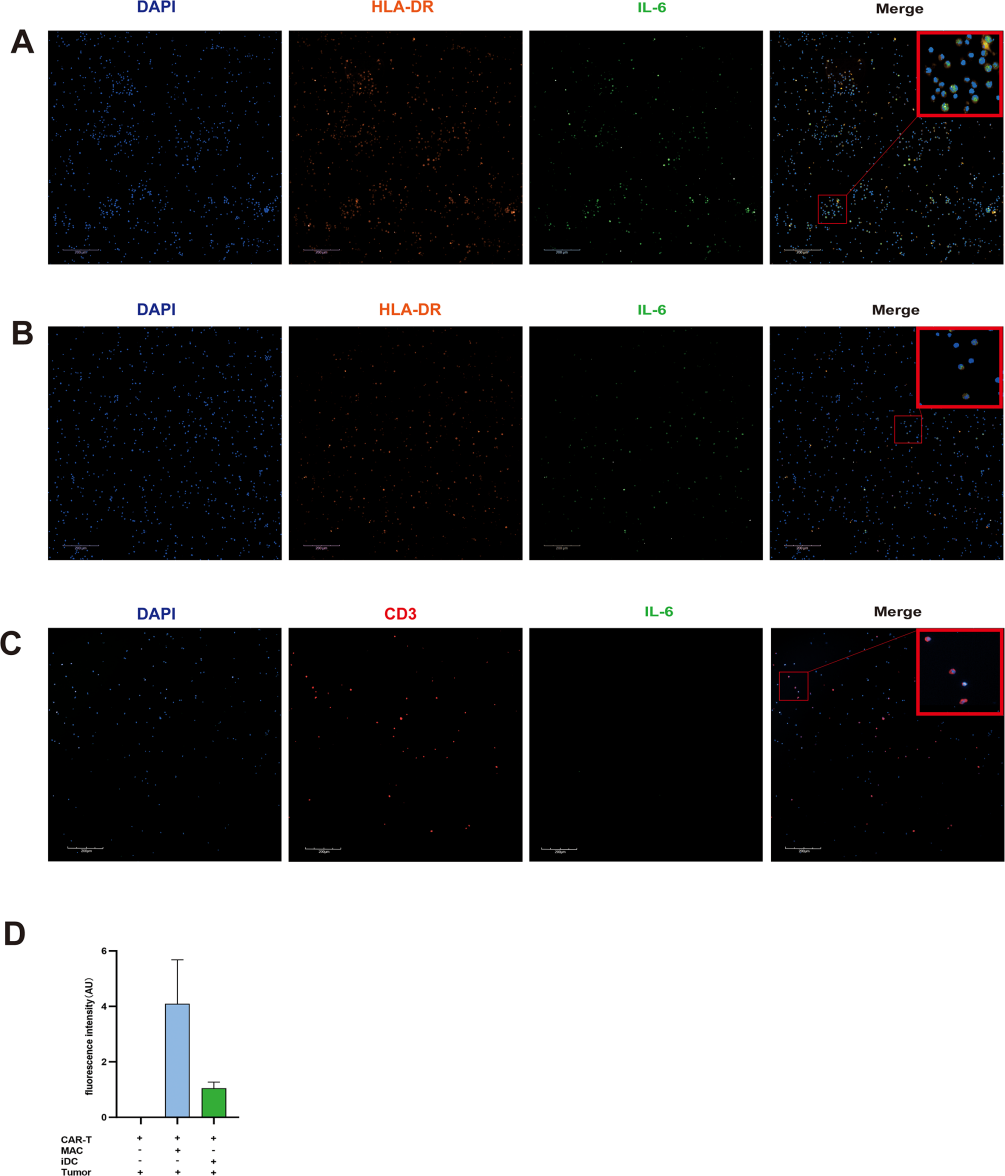
IL-6 is primarily derived from macrophages and iDCs in co-culture systems with CAR-T cells and tumor cells. **(A)** In co-cultures of CAR-T cells, macrophages, and tumor cells, IL-6 is mainly produced by macrophages. **(B)** In co-cultures of CAR-T cells, iDCs, and tumor cells, IL-6 is predominantly derived from iDCs. **(C)** Negligible IL-6 was detected when CAR-T cells were co-cultured with tumor cells alone. **(D)** Fluorescence intensity of IL-6. After co-culture of CAR-T cells, macrophages/iDCs, and tumor cells, IL-6 fluorescence intensity was detected, with the macrophage group showing the strongest fluorescence; n=3.

To investigate the cellular source of IL-6 during CRS, immunofluorescence staining was performed to detect co-expression of IL-6 and cell-specific surface markers on APCs and T cells. The results demonstrated that in triple-cell co-culture of CAR-T cells, APCs, and tumor cells, IL-6 was predominantly secreted by APCs (**Figures 7A, B**). In contrast, when only CAR-T cells and tumor cells were co-cultured, IL-6 was nearly undetectable (**Figure 7C**). Fluorescent intensity values demonstrated that IL-6 exhibited the strongest fluorescence when CAR-T cells, macrophages, and tumor cells were co-cultured (**Figure 7D**).The results indicated that APCs are essential for elevated IL-6 production.

### 3.5 APCs do not affect CAR-T cell cytotoxic function

**Figure 8:**
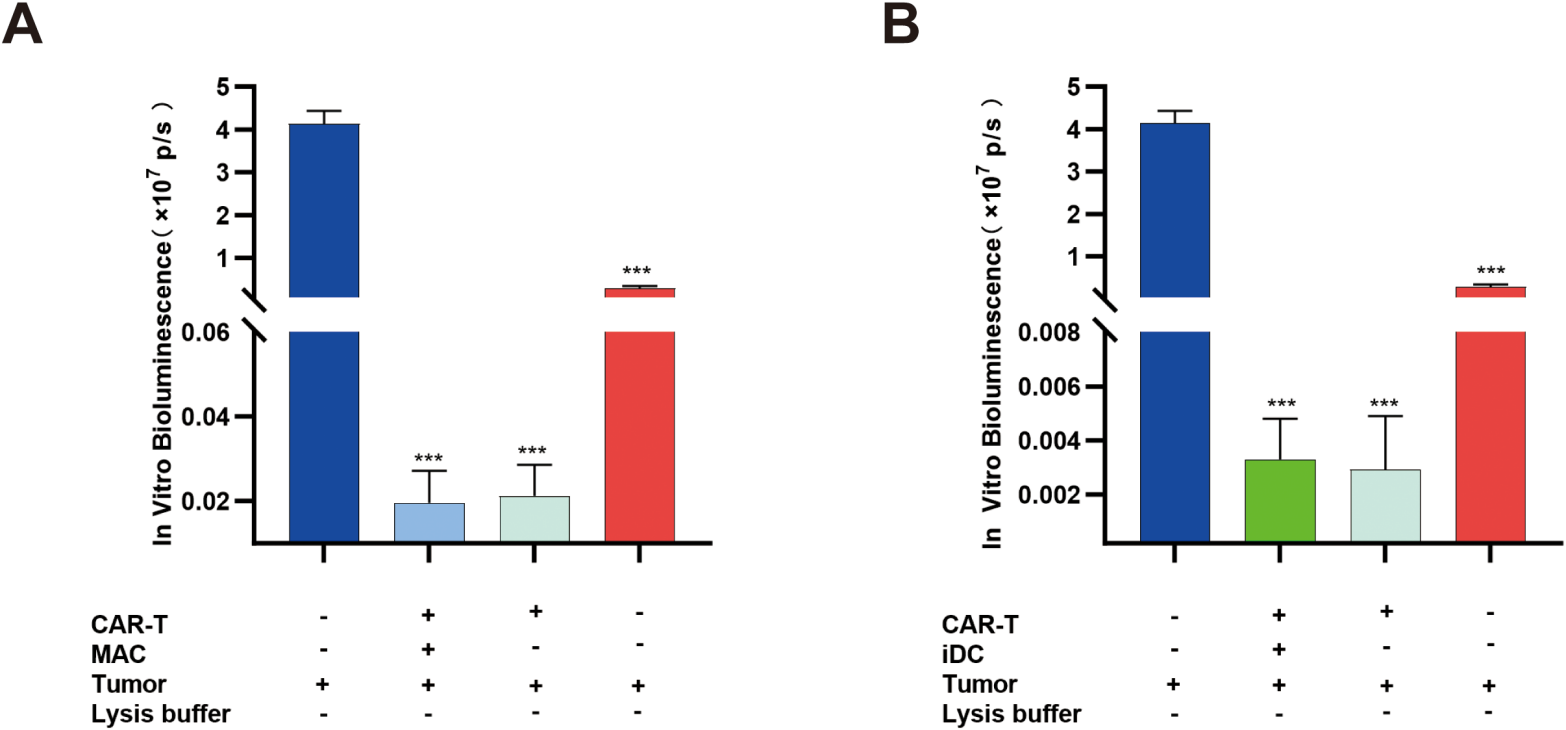
Macrophages and iDCs do not affect the function of CAR-T cells. **(A)** In the co-culture system of CAR-T cells, macrophages, and tumor cells, macrophages did not reduce the cytotoxicity of CAR-T cells. **(B)** In the co-culture system of CAR-T cells, iDCs, and tumor cells, iDCs did not reduce the cytotoxicity of CAR-T cells. ***P < 0.001; n=3.

To evaluate whether APCs or cytokines generated in the co-culture system influence CAR-T cell activity, residual tumor cells were analyzed by *in vitro* bioluminescence assay after co-culture. The results showed that the presence or absence of APCs did not alter the tumor cell killing capacity of CAR-T cells (**Figures 8A, B**).

## 4 Discussion

CAR-T-cell therapy has achieved significant advances in treating certain hematological malignancies. However, treatment-related adverse events such as CRS and immune effector cell-associated neurotoxicity syndrome (ICANS) remain life-threatening complications (Bachy et al., 2022). Therefore, identifying the potential CRS risk of CAR-T cells during early preclinical development is crucial for transitioning from preclinical studies to clinical trials. Currently, preclinical evaluation of CAR-T cells typically utilizes immunodeficient mouse models, including tumor xenograft models. However, the absence of a functional host immune system and inherent species differences limit their ability to fully recapitulate the cytokine cascade observed in human CRS. Although some mouse models have been developed for CRS prediction, their widespread application is constrained by the complexity of establishing humanized xenograft models (Norelli et al., 2018). Thus, there is an urgent need to develop reliable *in vitro* models for predicting CAR-T cell-induced CRS.

Conventional *in vitro* potency assays for CAR-T cells typically involve co-culturing with tumor cells and measuring cytotoxicity (Wang et al., 2023). In this study, CAR-T cells co-cultured with Nalm6-CBG cells exhibited potent tumor-killing activity, as evidenced by significantly reduced bioluminescence signals at E:T ratios ranging from 5:1 to 20:1. This cytotoxicity was accompanied by elevated IFN-γ levels. However, no IL-6 was detected under these experimental conditions. As a core cytokine in CAR-T-induced CRS, elevated IL-6 may induce vascular leakage, activate complement and coagulation cascades, and subsequently lead to disseminated intravascular coagulation and myocardial dysfunction (Tanaka et al., 2016). Consequently, the absence of IL-6 in the CAR-T and tumor cell co-culture system contrasts with the clinical presentation of CRS, indicating that this simplified binary culture model fails to adequately mimic human CRS pathogenesis.

CRS is driven by elevated cytokine levels following CAR-T cell infusion and target cell activation. However, assessing cytokine production in simple CAR-T and tumor cell co-cultures is insufficient for evaluating CRS risk, as CRS pathogenesis can be largely attributed to the role of endogenous monocytes that become activated and produce cytokines after CAR-T cell infusion (Giavridis et al., 2018). Studies have demonstrated that APCs can activate and expand CAR-T cells. DCs, which differentiate from monocytes, represent the most potent APC in the human body. They present antigens to T cells, stimulating T cell proliferation and cytokine release (Feng et al., 2023). Although CAR-T cells reduce the incremental kinetics required for MHC-driven endogenous T cell immune responses involving APCs (Ho et al., 2003), we hypothesized that enhanced killing capacity following antigen presentation by DCs would subsequently trigger more substantial cytokine release.

In this study, iDCs were first induced with or without TAA to generate tumor antigen-loaded or unloaded iDCs, which were then matured and co-cultured with CAR-T cells before assessing their tumor-killing capacity. Results demonstrated that DCs presenting tumor antigens to CAR-T cells significantly enhanced cytotoxic activity compared to the non-antigen loaded condition. This suggests that beyond direct CAR-mediated tumor antigen recognition through perforin/granzyme, Fas/FasL pathways, and cytokine secretion (Benmebarek et al., 2019), CAR-T cells may additionally recognize and kill tumor cells through antigen presentation by APCs. Furthermore, under these experimental conditions, IL-6 secretion was detected in both CAR-T cells alone and CAR-T-tumor co-culture groups, with significantly elevated IL-6 levels during tumor cell killing. Since the CAR-T cells collected after pre-culture with DCs contained residual DCs, and DCs are known sources of IL-6, the detected IL-6 likely originated from these DCs. As the cell density of TAA-CAR-T or no TAA-CAR-T increased, the corresponding DC numbers also rose, resulting in elevated IL-6 levels proportional to CAR-T cell density. The markedly increased IL-6 levels observed during tumor cell killing by TAA-CAR-T or no TAA-CAR-T cells correlate with previous findings that certain cytokines secreted during CAR-T-mediated killing, such as IFN-γ, can stimulate myeloid cells to release IL-6 and other cytokines (Xiao et al., 2021). These results align with findings from Singh et al., who reported elevated IL-6 levels when immature dendritic cells were incorporated into CAR-T and tumor cell co-culture systems (Singh et al., 2017). Consequently, this evidence indicates that DCs play a significant role in CRS pathogenesis.

However, in the human body, the sequential interactions between CAR-T cells, tumor cells, and myeloid cells (macrophages or DCs) are not strictly controlled. Previous studies have indicated that in addition to dendritic cells, macrophages also serve as major cellular sources of IL-6 during CRS (Giavridis et al., 2018). To better simulate CAR-T-induced CRS in humans, we established a triple-cell co-culture system containing CAR-T cells, macrophages or iDCs, and tumor cells, then measured the release of IFN-γ and IL-6. The results demonstrated that the inclusion of macrophages in the co-culture system did not significantly alter IFN-γ levels, whereas the addition of iDCs markedly enhanced IFN-γ production. Although IFN-γ can serve as a potency indicator for CAR-T cells, its precise biological role in responding to hematological malignancies remains incompletely defined. Studies by Singh et al. showed that any APC type (including macrophages and DCs) could elevate IFN-γ levels (Singh et al., 2017), whereas Bailey et al. demonstrated that IFN-γ knockout in CAR-T cells did not impair their cytotoxic function but reduced macrophage activation and associated cytokine toxicity (Bailey et al., 2022). These findings suggest that IFN-γ secretion may not be the primary mechanism underlying CAR-T cell cytotoxicity, though its excessive production can trigger substantial cytokine release from other cell types. In our experimental system, the incorporation of macrophages did not significantly enhance IFN-γ production. However, both macrophages and iDCs substantially elevated IL-6 levels, with macrophages playing a particularly critical role in IL-6 release; under these conditions, IL-6 levels reached 8,000 pg/mL. In a clinical study of CAR-T cell therapy (Huang et al., 2022), patients with grade 0–1 CRS showed IL-6 levels up to 500 pg/mL, while grades 2–3 peaked at 5,000 pg/mL. Therefore, under the experimental conditions of this study, the addition of macrophages to the co-culture system can simulate CRS of grade >3.

Macrophages and dendritic cells are key cell types mediating CRS. Although both originate from the myeloid lineage, they may play distinct roles in the CRS response. Macrophages are the primary source of the core cytokine IL-6 in CRS. Studies have shown that after CAR-T cell infusion, activated CAR-T cells secrete IFN-γ and GM-CSF, which strongly activate macrophages, leading to the massive production of inflammatory factors such as IL-6 and IL-1, directly driving systemic inflammatory responses (Giavridis et al., 2018; Norelli et al., 2018). On the other hand, iDCs, as professional APCs, primarily act as “initiators” and “amplifiers” of the inflammatory response in CRS. When iDCs uptake and process tumor antigens in the tumor microenvironment, they upregulate co-stimulatory molecules such as CD80 and CD86, providing strong activation signals (second signals) to CAR-T cells, thereby significantly amplifying CAR-T cell activation and IFN-γ production. This enhanced T-cell response further activates more myeloid cells, forming a positive feedback loop that exacerbates the cytokine storm (Ghasemi et al., 2024). In our study, similarly, after DCs uptook antigens and were co-cultured with CAR-T cells, they amplify CAR-T cell activation, enhance tumor-killing effects, and increase cytokine release. Additionally, when CAR-T cells, macrophages, and tumor cells are co-cultured, IL-6 levels significantly increase, whereas in co-culture systems with iDCs, IFN-γ levels significantly rise. This further demonstrates that macrophages and iDCs induce cytokine release through different mechanisms. Neither macrophage nor iDC co-culture affected CAR-T cell-mediated killing capacity. Immunofluorescence results further confirmed that the core CRS cytokine IL-6 originated predominantly from APCs. These findings suggest that during CAR-T cell therapy, modulating the activity of macrophages or DCs may help mitigate CRS-related toxicity. In summary, this study established a novel tri-cell co-culture model comprising CAR-T cells, myeloid cells (macrophages/dendritic cells), and tumor cells. By monitoring key CRS-associated cytokines (IFN-γ and IL-6), this *in vitro* system successfully recapitulates CRS induction by CAR-T cells under physiological conditions. While the established model offers distinct advantages for *in vitro* CRS evaluation, certain limitations remain. For instance, although IFN-γ and IL-6 levels may partially reflect the CRS-inducing potential of CAR-T cells, there are no definitive cytokine threshold standards for correlating with clinical CRS severity. Furthermore, CRS pathogenesis involves multiple cytokines beyond those measured, such as IL-1β, TNF-α, and GM-CSF. Therefore, future research will employ this co-culture model to compare CRS severity across CAR-T cells with different structural designs or targeting specificities. Additionally, multiplex cytokine profiling will be implemented to characterize comprehensive cytokine spectra, ultimately enhancing the predictive capacity of *in vitro* assays for evaluating CAR-T cell-associated CRS risks.

## 5 Conclusion

Macrophages and DCs play critical roles in CAR-T cell therapy-associated CRS. Based on the mechanistic understanding of CAR-T-induced CRS pathogenesis, this study established an *in vitro* model by co-culturing CAR-T cells, macrophages/dendritic cells, and tumor cells, enabling simulation of CRS through detection of the core cytokine IL-6. This co-culture model is time-efficient and experimentally straightforward, serving as a practical screening tool for early assessment of CAR-T-related CRS. Prior to complex *in vivo* studies, it allows preliminary high-throughput screening of multiple CAR constructs.

## Data Availability

The original contributions presented in the study are included in the article/supplementary material. Further inquiries can be directed to the corresponding authors.
